# Metabolic and Endocrine Toxicities of Mitotane: A Systematic Review

**DOI:** 10.3390/cancers13195001

**Published:** 2021-10-05

**Authors:** Marta Bianchini, Giulia Puliani, Alfonsina Chiefari, Marilda Mormando, Rosa Lauretta, Marialuisa Appetecchia

**Affiliations:** 1 Oncological Endocrinology Unit, IRCCS Regina Elena National Cancer Institute, Via Elio Chianesi 53, 00144 Rome, Italy; marta.bianchini@ifo.gov.it (M.B.); giulia.puliani@ifo.gov.it (G.P.); alfonsina.chiefari@ifo.gov.it (A.C.); marilda.mormando@ifo.gov.it (M.M.); rosa.lauretta@ifo.gov.it (R.L.); 2 Department of Experimental Medicine, Sapienza University of Rome, Viale Regina Elena, 324, 00161 Rome, Italy

**Keywords:** mitotane, endocrine toxicities, metabolic toxicities, adrenocortical cancer

## Abstract

**Simple Summary:**

This is, to our knowledge, the first systematic review conducted on the endocrine effects of mitotane, which aims to collect all available evidence in the literature and provide complete and useful information regarding the management of the endocrine and metabolic side effects of mitotane in clinical practice.

**Abstract:**

Despite the pivotal role of mitotane in adrenocortical carcinoma (ACC) management, data on the endocrine toxicities of this treatment are lacking. The aim of this systematic review is to collect the available evidence on the side effects of mitotane on the endocrine and metabolic systems in both children and adults affected by adrenal carcinoma. Sixteen articles on 493 patients were included. Among the adrenal insufficiency, which is an expected side effect of mitotane, 24.5% of patients increased glucocorticoid replacement therapy. Mineralocorticoid insufficiency usually occurred late in treatment in 36.8% of patients. Thyroid dysfunction is characterized by a decrease in FT4, which occurs within 3–6 months of treatment in 45.4% of patients, while TSH seems to not be a reliable marker. Dyslipidemia is characterized by an increase in both LDL-c and HDL-c (54.2%). Few studies have found evidence of hypertriglyceridemia. In males, gynecomastia and hypogonadism can occur after 3–6 months of treatment (38.4% and 35.6%, respectively), while in pre-menopausal women, mitotane can cause ovarian cysts and, less frequently, menstrual disorders. Most of these side effects appear to be reversible after mitotane discontinuation. We finally suggest an algorithm that could guide metabolic and endocrine safety assessments in patients treated with mitotane for ACC.

## 1. Introduction

Mitotane (o,p′-DDD), a synthetic derivative of the DDT (dichloro-diphenyl-trichloroethane) insecticide, is an adrenotoxic drug. It blocks the synthesis of hormonal corticosteroids through the inhibition of multiple enzymatic steps of steroidogenesis [1,2], but also has a direct cytotoxic effect on the adrenal cortex, inducing the degeneration of fasciculata and reticularis zones, dysfunction of mitochondria-associated membranes, and apoptosis in adrenal carcinoma cells [3,4,5].

For these anti-hormonal and anti-tumoural effects, its use was first approved in Cushing syndrome [6,7], and in the 1960s, it was introduced for the treatment of inoperable adrenocortical cancer (ACC) alone or in combination with systemic chemotherapy [8,9,10].

ACC is a rare tumour, with an incidence of 0.5–1 case per million inhabitants per year, with a slight female preponderance, and bimodal age distribution, with the larger peak between 40 and 50 years old [11,12,13].

This is an aggressive tumour with a 5-year overall survival of about 16–44%, which varies according to the stage of the disease [14,15,16]. Mitotane treatment is indicated in inoperable patients or after surgery for advanced, metastatic, or recurrent ACC [8,9,17,18]. Mitotane is recommended by the European Association of Endocrinology and the European Network for the Study of Adrenal Tumors (ENSAT), in their latest guidelines, as adjuvant therapy for at least two years after surgery, in patients with a high risk of recurrence (ENSAT stage III, or R1 resection, or Ki67 > 10%) [10]. 

Despite the pivotal role of mitotane in ACC management, treatment-related side effects are numerous and frequently occurring. The most common adverse events are gastrointestinal and hepatic disturbances (anorexia, nausea and vomiting, diarrhoea, hepatitis, or liver enzyme elevation) or neurological symptoms (confusion, sleepiness, ataxia, and dizziness) [19], which can lead to temporary or permanent discontinuation of the mitotane therapy [20,21,22,23].

Since side effects are more frequent and more severe in cases of high serum mitotane concentrations (above 20 mg/L), the monitoring of blood concentrations of mitotane is mandatory, with the general aim of reaching and maintaining it in the therapeutic range (between 14 and 20 mg/L) [10]. 

Regarding endocrinological toxicities, the most recognized and frequent one is adrenal insufficiency, which generally requires doses of glucocorticoid replacement therapy higher than those commonly used for primary adrenal insufficiency from other causes [21,24]. Less frequently, patients also develop mineralocorticoid insufficiency [25,26]. Mitotane can also alter thyroid function [21,27] and lipid metabolism, in particular, leading to hypercholesterolemia [28,29,30]. Current guidelines reported both of these latter side effects as very common, defined as a frequency of more than 1 in 10 treated patients [10]. In male patients, mitotane leads to hypogonadism (defined as common, occurring in 1 of 10–100 treated patients [10]) and gynecomastia (very common [10]). In females, mitotane can, less frequently, alter ovarian function [31]. 

Since ACC is a rare and aggressive disease, many studies focus on the anti-tumoural efficacy of mitotane [23,32], and only a small fraction of them, mostly isolated reports, evaluated the consequences of chronic mitotane treatment on the endocrine system. 

This systematic review aims to collect and discuss the available evidence on the endocrine and metabolic toxicities of mitotane and to assess the frequency, severity, time of onset, predisposing factors, required management, response to treatment, and eventual recovery of endocrine toxicity after mitotane discontinuation.

## 2. Materials and Methods

We performed this systematic review following the PRISMA guidelines [33]. 

### 2.1. Article Identification

We searched the PubMed database for studies reporting side effects of mitotane in patients affected by adrenal carcinoma. To screen all the possible articles, the search term used was “mitotane”. The last search date was 1 February 2021. No time restriction has been applied.

We included English-language studies on humans with any of the following designs: randomized clinical trials, prospective non-randomized trials, retrospective studies, and case series (including at least seven patients). 

The inclusion criteria were: (1) articles on mitotane used for the treatment of adrenal carcinoma; (2) articles reporting data on the endocrine and metabolic side effects of mitotane; (3) articles on both children and adults.

Exclusion criteria were: (1) non-original articles or case reports; (2) articles reporting only data on the efficacy of mitotane or non-endocrine side effects, without considering the related endocrine or metabolic toxicities. 

### 2.2. Article Selection

Each study was screened by its abstract and title, and potentially eligible studies were further assessed in detail by retrieving full-length articles. Each full-length article was independently reviewed by two separate authors following inclusion criteria. Two authors independently extracted data from the articles that met the inclusion criteria. A standardized form was used to extract the following information: study design, year of publication, number of patients enrolled, age at diagnosis, BMI, tumour staging, previous and concomitant treatments, data on mitotane treatment (dose, plasmatic concentration, duration), data on endocrine and metabolic side effects related to mitotane treatment (including the number of patients experiencing the side effects, severity, time of onset, predisposing factors), required management and treatment response, eventual mitotane discontinuation or dose reduction, and eventual recovery of the endocrine toxicities after therapy discontinuation.

## 3. Results

**Figure 1:**
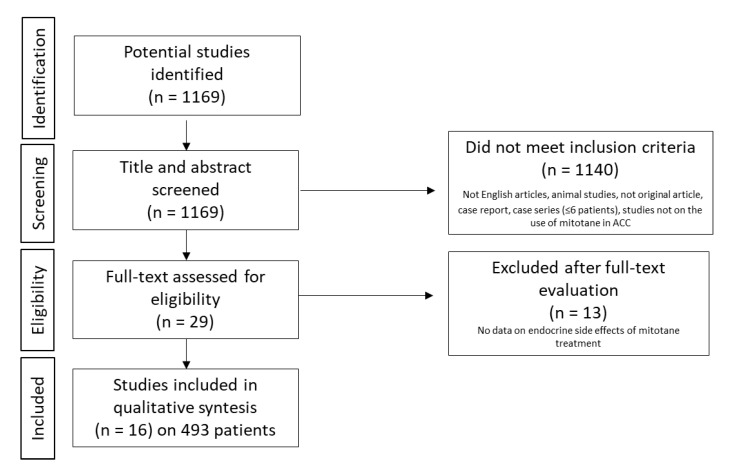
Flow-diagram of the systematic review. Abbreviation: ACC: adrenocortical carcinoma.

From the original 1169 articles, 29 were selected by title and abstract. After full-text examination, a total of 16 articles published between 1962 and 2020 were included in the systematic review (Figure 1). 

The main study characteristics, in addition to demographic and clinical features of patients, are summarized in Table 1. 

Female patients made up 63.3% (312/493) of all patients. In six studies, mitotane was used only as adjuvant therapy, while the other ten studies included patients treated for locally advanced or metastatic disease. Overall, the total number of patients treated in an adjuvant setting was 279 of 471 (59.2%). In six studies, mitotane was administered with concomitant chemotherapy (118 patients). Only three studies on adults reported BMI (range 17–54 kg/m^2^). The dosage of mitotane, when specified as a daily dose, ranged in most series from 1 to 5 g; the highest dosages of 10 and 20 g daily were reached in two older series [22,43]. Duration of treatment ranged from 1 week to 323 months. Tumour hormonal secretion was detected in 298 patients (67.6%). The frequencies of the metabolic and endocrine side effects are reported in Table 2. 

### 3.1. Adrenal Function

The adrenocytolytic effect of mitotane is known; therefore, adrenal insufficiency should be considered as an expected collateral event during treatment. Only three studies specified the need for increased steroid coverage due to serum ACTH level elevation, or to signs and symptoms of adrenal insufficiency, as an endocrine adverse event (13/53 patients, 24.5%) [19,22,40]. Mineralocorticoid replacement was administered in a total of 67 of 182 patients in six studies [21,29,31,34,40,43]. Moreover, another study reported the need for fludrocortisone replacement therapy, but the proportion of patients was unclear [22]. 

In a prospective study [21], serum cortisol levels decreased after 3 months from the beginning of mitotane therapy, and a significant inverse correlation between cortisol and mitotane serum concentration was found. The daily dose of cortisone acetate was 50–75 mg in 8/17 patients, 37.5 mg in 7/17 patients, and 25 mg in 2/17 patients [21], confirming that required hormone replacement doses are higher than normal [8,26,44,45]. This is due to the altered peripheral metabolism induced by mitotane, in particular, due to the increased clearance rate of glucocorticoids by liver enzymes [24,46]. Furthermore, the need for a higher glucocorticoid dose is also due to an increase in cortisol binding globulin (CBG) [47], which was also observed in this study, with a peak after 6 months. For this alteration of CBG, the serum cortisol assay is not always reliable in mitotane patients for the diagnosis and monitoring of adrenal insufficiency. Otherwise, salivary cortisol is unaffected by CBG levels and could be an index of free cortisol [48,49]; it is, therefore, theoretically better in patients treated with mitotane, even if the data are scanty. In fact, salivary cortisol was evaluated in only two studies, which reported low salivary cortisol levels [21,35].

Besides the classical pathogenic mechanisms of mitotane-induced adrenal insufficiency, another mechanism that involves pituitary secretion of ACTH has been recently proposed. Indeed, Gentilin et al. [50] showed that in mouse cell lines, mitotane reduced pituitary cell viability and ACTH secretion. Interestingly, this is in accordance with the ability of mitotane to reduce pituitary TSH production. Reimondo et al. confirmed these results by in vivo study. Basal ACTH levels of 16 patients treated with mitotane were lower than the levels of 10 patients affected by primary adrenal insufficiency, and ACTH was also less responsive to corticotrophin-releasing hormone (CRH) stimulation in patients treated with mitotane [35].

Poirier et al. evaluated the recovery of adrenal insufficiency in 23 patients after mitotane discontinuation: 78.3% achieved a complete recovery of hypothalamic-pituitary-adrenal (HPA) axis with a median of 2.8 years from mitotane last dose; 13% of patients had partial recovery, with normal serum cortisol concentrations but with symptoms of adrenal insufficiency that prevented corticosteroid replacement withdrawal. Lastly, only 8.7% of patients never achieved recovery of the HPA axis. Unfortunately, the authors failed in the identification of any predictors of the HPA axis recovery [34]. Accordingly, another study reported that 15 out of 24 patients tested with ACTH stimulation test showed a recovery of adrenal function 6 months after the mitotane therapy discontinuation [29]. 

Concerning mineralocorticoid insufficiency, in the prospective study by Daffara et al. [21], 65% of patients started mineralocorticoid replacement for hypotension or dizziness after 6–9 months, even if the authors did not find significant changes in aldosterone and plasma renin activity (PRA) serum concentration. This probably suggests that the glomerulosa zone is less sensitive to the cytotoxic effect of mitotane than the fasciculata zone; this is in line with a previous study that reported a relative sparing of this cortical zone [20]. This is also in agreement with an older study that reported a later development of mineralocorticoid insufficiency over adrenal insufficiency, appearing several months after starting mitotane therapy [25].

However, the results about mineralocorticoid impairment during mitotane treatment are conflicting. In fact, Basile et al. reported increased PRA and reduced aldosterone levels after 6 months of mitotane treatment, similar to cortisol and ACTH serum levels. Moreover, after the introduction of fludrocortisone, PRA values decreased only in some patients, suggesting an insufficient replacement. Authors have hypothesized that mitotane can also enhance fludrocortisone metabolism, as in the glucocorticoid one [31]. 

Glucocorticoid and mineralocorticoid deficiency are also observed in children treated with mitotane; in fact, in the prospective study by Zancanella et al. [40], adrenal insufficiency was the cause of death in one patient, while 5 of 11 patients presented with signs and symptoms of adrenal insufficiency. Therefore, the authors recommended corticosteroid replacement therapy also in children, using 10–15 mg/m^2^ of prednisone daily in 2–3 doses or equivalent doses of dexamethasone, associated with 0.15–0.2 mg of fludrocortisone daily, always increasing dosages during periods of stress [40]. 

### 3.2. Thyroid Function

Four studies reported hypothyroidism as an adverse event, identified by reduction of free thyroxine (FT4) levels, occurring in 68 of 141 patients whose thyroid function was measured [21,22,29,31]. In three of these studies, FT4 changes occurred after 3–6 months of treatment, while no significant change of thyroid-stimulating hormone (TSH) concentration was recorded [21,29,31]. Fifty-four patients were treated with levothyroxine. Only one study reported increased thyroid hormone replacement therapy in patients already on treatment after starting mitotane treatment [40]. 

Usually, 3–6 months of thyroxine treatment are necessary to normalize thyroxine (T4) and FT4 levels, and total T4 increases after mitotane discontinuation [29].

Available studies reported a reduction in FT4 serum levels, with no change in free triiodothyronine (FT3) and TSH. Initially, it was hypothesized that the reason could be a change in the plasma transport of the thyroid hormone through the increase of thyroxine-binding globulin (TBG) [51,52]. However, available data showed that not only the total T4 but also the free form (FT4) is affected. Therefore, other possible explanations have been advocated for. Mitotane can enhance the conversion of T4 to T3 by deiodinase, as confirmed by the FT3/FT4 ratio being in the upper reference range [26], which is a frequently observed compensatory mechanism in patients affected by hypothyroidism. Moreover, mitotane could inactivate TSH, for example, through abnormal glycosylation [53,54]. In fact, in vitro studies show that mitotane reduces both the expression and secretion of TSH in a dose-dependent manner and blocks the TSH response to thyrotropin-releasing hormone (TRH), determining central functional hypothyroidism [55]. In 2015, Russo et al. [26] confirmed by in vivo study that mitotane-induced hypothyroidism is very similar to central hypothyroidism; in fact, the authors found that TSH increased after thyrotropin-releasing hormone (TRH) administration, which was similar in 5 females patients affected by ACC and treated with mitotane, and in 10 patients affected by central hypothyroidism.

### 3.3. Dyslipidemia

Therapy with mitotane also showed effects on the lipid profile in 104/196 patients (53%) in seven studies. Four studies have found an increase in total cholesterol during mitotane treatment for a total of 54/110 patients (49%), mainly due to an increase in high-density lipoprotein cholesterol (HDL-c) and low-density lipoprotein cholesterol (LDL-c). 

Dyslipidemia, and in particular hypercholesterolemia, is one of the best-known side effects of mitotane. One of the first studies on mitotane endocrine and metabolic side effects was published by Molnar and colleagues in 1962 and reported a high prevalence of hypercholesterolemia in a series of treated patients [43]. Although the doses of mitotane used (5–12 g daily) were certainly higher than the dosages currently recommended, the authors’ conclusions are still valid in light of the most recent studies: the effect on cholesterol is, in many cases, reversible with the withdrawal of mitotane and tends to recur when patients resume treatment [56].

Concerning pathogenic mechanisms, it is known that mitotane increases LDL-c through stimulation of hydroxymethylglutaryl-coenzyme A (HMG-CoA) reductase, the rate-limiting enzyme in cholesterol synthesis [30,57]. On the contrary, the mechanism involved in the HDL-c increase is less clear. It is hypothesized that mitotane induces the activity of the liver cytochrome P450 stimulating HDL synthesis [58], but also the estrogen-like activity of mitotane may be involved [59,60]. Furthermore, mitotane determines the dysfunction of scavenger receptor class B member 1 (SR-B1), localized in the liver and steroidogenic organs, that are involved in the selective uptake of HDL [61]. In addition, mitotane inhibits CYP11A1, reducing the conversion rate of cholesterol to pregnenolone [24]. Finally, hypothyroidism, another mitotane-induced endocrine toxicity, theoretically increases cholesterol levels. 

In the retrospective study by Shawa et al. [28], both HDL-c and LDL-c concentrations increased significantly (mean change, 33.1 mg/dL and 45.7 mg/dL, respectively), and the median time to peak was 234.5 days for HDL-c and 173.5 days for LDL-c.

Lipid alterations also impact triglyceride levels. Three studies reported data on patients who develop dyslipidaemia, without distinguishing between alterations in triglycerides, cholesterol, or both, for a total of 78/148 patients (52.7%) [28,29,31]. 

The effect of mitotane on triglycerides is controversial because a significant change was reported in some studies [31], while in others, the effect of mitotane was evident only on cholesterol levels [21]. Shawa et al. found that triglycerides levels increased significantly (mean change = 67.7 mg/dL) considering all patients in the study; however, the increase was not significant after the exclusion of patients who were on lipid-lowering drugs at baseline [28]. This could explain the conflicting results regarding the increase in triglycerides observed between studies. Mitotane levels are positively correlated with HDL-c levels, which is not surprising since most of the adverse events of mitotane are dose-dependent. This positive correlation was not confirmed with LDL-c or triglycerides levels, probably because low serum levels of mitotane are enough to stimulate the HMG-CoA reductase activity. Finally, the authors did not find any clinical predictors of HDL change between BMI, sex, cortisol secretion, or alpha-lipoproteinemia levels at baseline [28].

Accordingly, Vikner et al. demonstrated that total cholesterol, LDL-c, HDL-c, and triglyceride serum levels increased significantly, in particular after 6 months, and a positive correlation was observed only for mitotane serum levels and HDL-c concentrations. In this study, patients were treated with simvastatin, atorvastatin, and rosuvastatin, determining a reduction of total cholesterol and LDL-c after 3 months. After 12 months of discontinuation of mitotane therapy, a significant improvement in the lipid profile was observed in 30 patients, although about a third of them (13 patients) continued to take statin therapy, attesting a failure to recover from dyslipidaemia [29]. In conclusion, this study [29] shows that LDL-c increased faster than HDL-c within 6 months in all cases and that the increase in HDL levels should be considered in the decision-making process (for its cardioprotective effect) regarding the start of lipid-lowering therapy.

The onset of metabolic adverse events seems to not be correlated with the achievement of mitotanemia ≥ 14 mg/dL (target value); in fact, Basile et al. reported that statin therapy was also necessary for patients with lower serum mitotane levels [31]. 

Physicians should be careful in choosing the best lipid-lowering agent, as mitotane induces CYP3A4, which is involved in the metabolism of many statins. For this reason, guidelines recommend pravastatin or rosuvastatin for the treatment of mitotane-induced dyslipidaemia [10]. This recommendation, however, stems more from the pharmacokinetic considerations than from low clinical data on the use of lipid-lowering agents in patients treated with mitotane.

One study reported that, in patients treated with mitotane and rosuvastatin, the percentage reduction in LDL-c levels was lower than that reported in the general population, suggesting that mitotane may affect the metabolism of rosuvastatin independently of CYP3A4 [31].

### 3.4. Male Hypogonadism and Gynecomastia

Most of the studies reported, among mitotane toxicity, symptoms of male hypogonadism, mainly sexual symptoms, erectile dysfunction, and gynecomastia. Frequency of gynecomastia is 38 out of 99 male patients (38.4%) [19,21,22,29,38,39,40,41,42], while male hypogonadism was observed in 26 of 73 patients (35.6%) [21,22,29,31]. One study [36] reported that 4 of 15 men experienced symptoms of hypogonadism and/or gynecomastia without providing single frequencies. 

A biochemical evaluation was performed in three of these studies [21,29,31]. In a prospective study conducted from 1999 to 2005, the hormonal status was evaluated in 7 male patients at baseline and periodically every 3 months for a year [21]. A biphasic behaviour of total testosterone was observed, with a not statistically significant increase at 3 and 6 months followed by a rapid decrease. Free testosterone levels significantly decreased during mitotane therapy, while sex hormone-binding protein (SHBG) levels increased significantly, reaching a peak after 3 months of treatment. 

A significant increase in SHBG levels was also observed in another study on 35 male patients [31], occurring in the first 6 months, associated with a significant reduction in free testosterone values and a non-significant increase in total testosterone. In both studies, luteinizing hormone (LH) and follicle-stimulating hormone (FSH) levels did not change significantly [21,31]. 

In another recent retrospective study, Vikner et al. [29] confirmed that mitotane significantly increases plasma SHBG, but in contrast to previous studies, it also causes an increase in LH and total testosterone without significant changes in free testosterone levels. 

Testosterone treatment was prescribed in 20 patients in three studies [21,29,31] with discordant clinical benefits. Vikner et al. started testosterone therapy in four symptomatic men with limited success for their symptoms [29]. On the contrary, an improvement in strength, mood, and sexual desire was reported in the study by Daffara et al. in 4 of 7 men. Both studies reported, along with testosterone replacement, a worsening of gynecomastia (globally, three patients), probably due to an increased testosterone conversion to oestradiol [21].

Basile et al. reported no data on the clinical effects of treatment but evaluated the onset of male hypogonadism (defined as the need for testosterone treatment). Hypogonadism occurs when mitotane serum concentrations are at least 14 g/dL, after an average time of 33 months of therapy (range 5–78 months) [31]. 

After treatment, one study reported a significant increase in total and free testosterone levels after 6 months of replacement therapy without changes in gonadotropin levels [31], and another showed no changes in LH and SHBG values during testosterone treatment [29]. Moreover, SHBG decreased 10 months after mitotane discontinuation [29,31].

The mechanisms hypothesized to explain hypogonadism in this context vary. The induction of SHBG synthesis could explain the reduction in free testosterone levels [62]; in addition, limited data in the literature support the direct toxic damage of mitotane at the testicular level [63]. Another proposed mechanism is the inhibition of the 5α reductase activity, responsible for the conversion of testosterone into the more potent androgen dihydrotestosterone (DHT) [24]. In other words, low DHT levels could be responsible for hypogonadal symptoms, and specifically, for gynecomastia, because of the enhanced conversion of testosterone to 17β-oestradiol [64]. Failure to convert testosterone to DHT could also cause the observed clinical inefficiency of the testosterone replacement therapy [29]. 

The assessment and treatment of hypogonadism are often underestimated in patients receiving mitotane treatment. The total testosterone measurement alone could not correctly diagnose hypogonadism due to the concomitant increase in SHBG: free testosterone evaluation is therefore required, starting at least within 6 months of mitotane therapy.

Considering the quality of life of patients affected by cancer, testosterone treatment should be considered for the potential benefit on asthenia, mood, and sexual life, which, as reported in an oncological setting, could become a more relevant issue over time [65]. Careful treatment monitoring is mandatory, considering that testosterone replacement therapy could be complicated by an increased rate of gynecomastia.

### 3.5. Ovarian Cysts

The development of ovarian cysts in pre-menopausal women treated with mitotane has been described in few studies. The study by Selenave and colleagues, which first reported this finding, included women who received mitotane for adjuvant treatment of ACC (*n* = 13), but also for the treatment of Cushing disease (CD) (*n* = 8). Ovarian macrocysts were detected in these patients after a median of 11 months of mitotane exposure (range 3–36 months). In two patients with CD, regression of the cysts was observed 3–4 months after mitotane discontinuation. The diagnosis was made in 67% of patients for the presence of symptoms or signs, like pelvic pain, abnormal bleeding, or oligo/amenorrhea. The cysts were complicated in two cases by adnexal torsion and haemorrhagic cyst rupture, requiring emergency surgery [37].

Another small case series [66] included five women of reproductive age diagnosed with ACC stages III-IV under adjuvant mitotane treatment. Ovarian cysts were detected during the follow-up by computed tomography (CT), Magnetic Resonance Imaging (MRI), or ultrasonography and showed no suspicious ultrasound features. Carbohydrate antigen 125 (Ca-125) levels were in the normal range. As previously described, after treatment interruption, the cysts regressed. One patient underwent salpingo-oophorectomy, and the histological examination showed a benign mass. Only 2 of 5 women became amenorrhoeic during mitotane treatment, and normal cycles returned after therapy discontinuation. In both studies, the cysts’ diameter ranged from 20 mm to 100 mm [37,66].

In a recent study in the adjuvant setting, ovarian cysts (at least 2 cm) were not observed in postmenopausal women, while a prevalence of 65.4% was reported in women of childbearing potential after a mean of 8 months from the start of mitotane therapy. Menstrual irregularity was observed in only 30.8% of patients. When evaluated, the occurrence of ovarian cysts appeared to be independent of the serum concentration of mitotane [31].

The mechanisms leading to the formation of ovarian cysts are not clear. In a study by Abrahamsson et al., a reduction of progesterone release was observed in cultures of human granulosa cells exposed to mitotane in vitro, with an apoptotic effect on the steroid-producing cells of the ovary [66]. In the study by Selenave and colleagues, an important decline in testosterone and delta-4-androstenedione was observed during mitotane therapy, which was not fully explained by the decline in adrenal dehydroepiandrosterone sulfate (DHEAS) [37]. These observations suggest that mitotane can interfere with ovarian steroidogenesis, as already known for adrenal steroidogenesis. Cyst formation could also depend on an increased pituitary secretion of gonadotropins, resulting from impairment of ovarian steroid production. However, only one study reported elevated gonadotropin levels during mitotane therapy [37]. 

Another proposed explanation is that mitotane acts on local ovarian factors, for example, cytokines, leading to altered sensitivity to gonadotropins [66].

One study reported mild hyperprolactinemia in 3 out of 10 women treated by mitotane without describing associated menstrual irregularity or hormonal profile alterations [21]. 

## 4. Conclusions

Mitotane treatment is unfortunately associated with many side effects. Some of them are well known and are a direct consequence of the drug’s mechanism of action, while other toxicities are less known. Unless understudied, endocrine side effects are frequent and could affect the patients’ quality of life. However, they can be successfully treated if promptly diagnosed, and this could avoid mitotane discontinuations and ameliorate treatment adherence.

The evaluation of serum levels of mitotane is a fundamental part of good clinical practice, levels above 14 mg/dL predict treatment efficacy, but a lower concentration does not guarantee treatment safety.

Adrenal insufficiency is very frequent and occurs earlier than mineralocorticoid insufficiency; therefore, it is recommended that complete glucocorticoid replacement be initiated simultaneously with the start of mitotane therapy, considering that severe hypoadrenalism is a life-threatening condition. This adrenal insufficiency could be reversible, but the recovery of the hypothalamic-pituitary-adrenal (HPA) axis has not been described prior to two years after mitotane discontinuation.

During mitotane treatment, patients may develop thyroid dysfunction in terms of decreased FT4, which is the consequence of central hypothyroidism, occurs in 3–6 months, and is usually reversible after mitotane discontinuation. 

Dyslipidaemia, common during mitotane treatment, is characterized by a simultaneous increase in both LDL-c and HDL-c. An increase of triglycerides has also been observed in a lower percentage of patients. The onset of dyslipidaemia occurs approximately 6 months after starting mitotane therapy. The lipid profile improves, in many cases, after discontinuation of mitotane therapy, but recovery is not complete in some patients who need statin therapy even after mitotane withdrawal. 

In males, gynecomastia and hypogonadism can occur after 3–6 months of treatment. Testosterone replacement therapy can improve hypogonadism but can also worsen gynecomastia. 

In pre-menopausal women, mitotane can cause reversible ovarian cysts and, less frequently, menstrual disorders.

**Figure 2:**
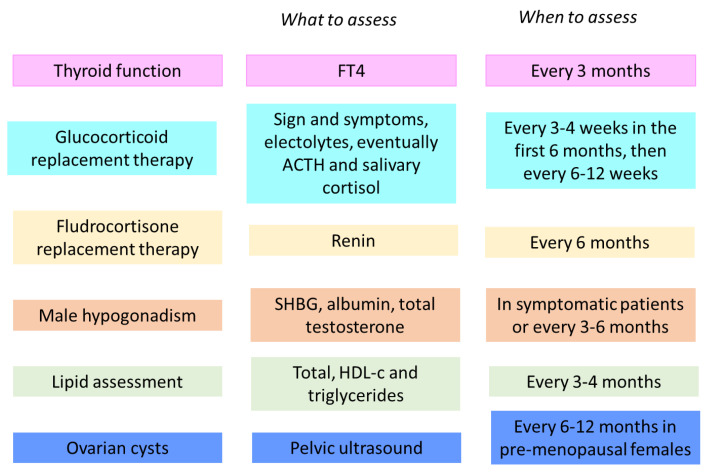
Algorithm for metabolic and endocrine safety assessment in patients treated by mitotane.

Basing on the collected evidence and the available guidelines, we suggest an algorithm that could guide metabolic and endocrine safety assessment in patients treated by mitotane, which is reported in Figure 2.

Assessment of adequacy of glucocorticoid replacement therapy should be based on signs and symptoms, a significant change in body weight, postural blood pressure, or electrolyte plasma levels [67]. Clinical examination should be performed every 3–4 weeks in the first 6 months and then every 6–12 weeks [10]. Assessment of ACTH and cortisol is usually not required. However, it is important to note that the determination of ACTH and salivary cortisol should be preferred to serum cortisol. Renin levels could be monitored every 6 months to guide the decision of starting fludrocortisone replacement therapy [10]. The possibility of late recovery of the adrenal function suggests performing a stimulatory test, as the short corticotropin test (250 μg), after 3–6 months from treatment discontinuation and, if insufficiency persistence, repeat the test annually. 

FT4 evaluation should be used instead of TSH assessment both for hypothyroidism diagnosis and for levothyroxine treatment dose adjusting, in line with the American Thyroid Association’s (ATA) guidelines for central hypothyroidism [68]. After baseline evaluation, we suggest assessing FT4 every 3 months, according to the ENSAT guidelines [10]. In the event of changes in thyroid function, clinicians will customize the timing of thyroid monitoring.

Lipid assessment, by total and HDL cholesterol and triglycerides, should be performed every 3–4 months [10]. A complete assessment of the patient’s cardiovascular risk should guide treatment, starting with lipid-lowering agents [69], while also considering the balance between HDL-c and LDL-c, which are both increased by mitotane therapy. Drugs metabolized by CYP3A4 should be avoided.

In male patients, it is fundamental to evaluate signs and symptoms of hypogonadism, as reduced sexual desire and activity, loss of axillary and pubic hair, decreased spontaneous erections, and erectile dysfunction [70,71]. In symptomatic patients or every 3–6 months, SHBG, albumin, and total testosterone should be evaluated to calculate free testosterone and promptly start replacement therapy.

Finally, for pre-menopausal females, a careful history of gynaecological symptoms is recommended, and pelvic ultrasound is suggested after 6–12 months from treatment start, and then annually, for early detection of ovarian cysts. Hormonal assessment should be reserved for limited cases, for example, in case of menstrual irregularities, considering that the mitotane treatment rarely affects hormonal levels.

## Figures and Tables

**Table 1:** Demographic and clinical features of patients. Abbreviations: F, female; M, male; BMI, body mass index; A, adjuvant; M, metastatic; U, unresectable; CHT, chemotherapy; am, aminoglutethimide; R, retrospective; P, prospective; CS, case series.

First Author, Year (Ref.)	Study Design	Patients Number Total (F/M)	BMI (Range)	Age (Range)	Mitotane (A/U/M)	Concomitant CHT	Median Dosage of Mitotane	Median Concentration of Mitotane (mg/L)	Duration of Therapy Mean Months (Min-Max)	Stage at Diagnosis n (%) (I-II-II-IV)	Functional Status n (%)
Poirier J, 2020 [34]	R	23 (15/8)	–	41 (18–73)	A: 100%	No	– 2–4.5 g ^d^	19.4 (8.1–27.1) (maximum dose)	34 (24–85)	I: 5/23 (21.7) II: 14/23 (60.9) III: 4/23 (17.4)	8 (34.8) 9 NA
Basile V, 2020 [31]	R	74 (39/35)	–	46 (18–77)	A: 100%	No	–	–	40 (12–195)	10 (13.5) 56 (75.7) 8 (10.8) 0 (0)	38 (51.4)
Vikner ME, 2020 [29]	R	50 (35/15)	–	54 (18–79)	M: 100%	21 (42%)	3.5 g (-) ^c^	13.5 (9.7–16.2) after 12 months	11 (5–26)	0 (0) 0 (0) 26 (52) 24 (54)	30 (60)
Reimondo G, 2017 [35]	P	16 (6/10)	–	52 (35–70)	A: 100%	No	2 g (1–4.5)	13.5 (5.7–24.3)	31 (12–63)	I–II–III: 16/16 (100)	5 (31.25)
Maiter D, 2016 [36]	R	34 (21/13)	–	46.5 (16–82)	A: 35%	21 (62%)	3.0 g (0.5–7.5)	–	20 (1–323)	1 (2.9) 5 (14.7) 10 (29.4) 18 (53)	22 (65)
Salenave S, 2015 [37]	R	21 (21/0) 13 ACC 8 CD	29 (17–47)	33 (18–45)	A: 100%	–	2 g (1–3.5) ^e^	14.3 (5–20.5) after 12 months	–	–	6 (46.2)
Shawa H, 2013 [28]	R	38 (26/12)	34.6 (20.3–54) ^a^	52.5	A: 32% M: 66% U: 2%	10 (26%)	–	–	9.7 (1–47.5)	2 (5.3) 18 (47.4) 13 (34.2) 5 (13.2)	15 (39.5)
Mauclere- Denost S, 2012 [38]	P	22 (14/8)	23.5 (19–31)	59 (25–73)	A: 13 (59%) M/U: 9(41%)	–	405 g (157–546) ^b^	–	–	0 (0) 6 (27) 11 (50) 5 (23)	15 (68)
Daffara F, 2008 [21]	P	17 (7/10)	–	36 (22–58)	A: 100%	No	4 g (2–4)	< 20	at least 12	1 (6) 12 (70) 3 (18) 1 (6)	11 (65)
Terzolo M, 2007 [39]	R	47 (36/11)	–	42 (18–67)	A: 100%	No	– (1–5 g)		29 (6–164)	3 (6.4) 27 (57.4) 11 (23.4) 6 (12.8)	24 (51.1)
Zancanella P, 2006 [40]	P	11 (8/3)	17.8 (12.7–25.2)	2–11.3 ^a^	M/U: 100%	10	3.09 g/m^2^ (-)		– 5–25	1 (9.1) 2 (18.2) 3 (27.3) 5 (45.4)	11 (100)
Terzolo M, 2000 [19]	P	8 (4/4)	22 (18–26)	52 (45–62)	A: 2 (25%) M: 6 (75%)	2: previous CHT	363 g (283–387)		9 (8–40)	I–II: 2 (25) III: 1 (12.5) IV: 5 (62.5)	4 (50)
Khan TS, 2000 [41]	P	40 (26/14)	–	44 (20–69)	A: 17 M: 12 U: 11	All: streptozocin	3.0 g (1–4)		5 (1 week–20 months)	I–II: 25 (62.5) III–IV: 15 (37.5)	22 (55)
Luton JP, 1990 [42]	R	59 (38/21)	–	46 (6–81)	A: 23 U: 14 NA: 22	16 (CHT+ am)	7 g (3–20)		10.5 (1–96)	I–II–III: 62/88 (70) IV 26/88 (30)	83 (79)
Van Slooten H, 1984 [22]	R	34 (19/15)	–	46.6 (20–71)	M: 100%	No				IV 34 (100)	17 (50)
Molnar GD, 1962 [43]	CS	7 (5/2)	–	40 (23–58)	M: 100%	No	– (4–10 g)		4.5 (1.5–22)	IV 7 (100)	6 (85.7)

^a^, on 26 patients; ^b^, cumulative dose after 3 months; ^c^, after 6 months; ^d^, maximal daily dose; ^e^, ACC cohort; NA, not available. Study included 16 studies, a total of 493 paediatric and adult patients were included, with ages ranging from 2 to 81 years. Fourteen studies enrolled only adult patients, one study only children, and another study included both (10 patients up to 20 years and 95 patients over 20 years of age).

**Table 2:** Frequencies of endocrine toxicities. Data are reported as *n* (%).

First Author, Year [Ref]	Adrenal Insufficiency *	Mineralocorticoid Deficit	Hypothyroidism	Gynecomastia	Male Hypogonadism	Ovarian Cysts	Hypercholesterolemia	Hypertriglyceridemia
Poirier J, 2020 [34]	-	8/23 (34.8)	-	-	-	-	-	-
Basile V, 2020 [31]	-	24/74 (32.4)	25/69 (36.2)	-	12/35 (34.3) ^a^	17/39 (43.6)	35/70 (50) ^a^	
Vikner ME, 2020 [29]	-	10/50 (20)	22/50 (44)	4/15 (26.6)	4/15 (26.6)	-	39/50 (78)	
Maiter D, 2016 [36]	-	-	-	4/13 (30.7)		-	-	-
Shawa H, 2013 [28]	-	-	-	-	-	-	4/28 (14.3) ^a^	
Mauclere-Denost S, 2012 [38]	-	-	-	4/8 (50)	-	-	-	-
Daffara F, 2008 [21]	-	11/17 (65)	12/13 (92.3)	5/7 (71.4)	4/7 (57.1) ^b^	-	8/17 (47)^a^	-
Terzolo M, 2007 [39]	-	-	-	4/11 (36.3)	-	-	-	-
Zancanella P, 2006 [40]	6/11 (54.5)	11/11 (100)	1/11 (9.1)	3/3 (100)	-	-	-	-
Terzolo M, 2000 [19]	3/8 (37.5)	-	-	1/4 (25)	-	-	7/8 (87.5)	7/8 (87.5)
Khan TS, 2000 [41]	-	-	-	4/14 (28.5)	-	-	-	-
Luton JP, 1990 [42]	-	-	-	7/21 (33.3)	-	-	-	-
Van Slooten H, 1984 [22]	4/34 (11.8)	-	9/9 (100)	6/16 (37.5)	6/16 (37.5) ^b^	-	7/16 (43.7)	-
Molnar GD, 1962 [43]	-	3/7 (42.8)	-	-	-	-	4/7 (57.1)	-
Overall	13/53 (24.5)	67/182 (36.8)	69/152 (45.4)	38/99 (38.4) ^c^	26/73 (35.6) ^c^	17/39 (43.6)	26/48 (54.2)	7/8 (87.5) ^d^

*: defined as increase in glucocorticoid replacement therapy because of ACTH elevation or signs/symptoms of adrenal insufficiency; a: defined as treatment necessity; b: defined as impotence; c: calculated without considering Maiter D et al.; d: calculated without considering Basile V et al., Vikner ME et al., Shawa et al.
